# Complete Genome Sequence of a Novel Porcine Parvovirus (PPV) Provisionally Designated PPV5

**DOI:** 10.1128/genomeA.00021-12

**Published:** 2013-01-15

**Authors:** Chao-Ting Xiao, Patrick G. Halbur, Tanja Opriessnig

**Affiliations:** Department of Veterinary Diagnostic and Production Animal Medicine, College of Veterinary Medicine, Iowa State University, Ames, Iowa, USA

## Abstract

A new porcine parvovirus (PPV) was identified from pig lung tissues in the United States. It is most closely related to the recently identified PPV4 with overall genomic identities of 64.1 to 67.3%. Unlike PPV4, this virus lacks the additional open reading frame 3 (ORF3) and has a much longer ORF2. The name PPV5 is provisionally proposed.

## GENOME ANNOUNCEMENT

Parvoviruses have a small, nonenveloped, single-stranded DNA genome of 4 to 6.3 kb (1). The family *Parvoviridae* is comprised of two subfamilies, *Parvovirinae* and *Densovirinae*. The *Parvovirinae* subfamily is further subdivided into five genera: *Parvovirus*, *Erythrovirus*, *Dependovirus*, *Amdovirus*, and *Bocavirus* (1). Parvoviruses usually possess two major gene cassettes (open reading frame 1 [ORF1] and ORF2); the ORF1 encodes the nonstructural proteins (NS) required for transcription and DNA replication, and the ORF2 encodes the structural proteins of the capsid (1). Five different groups of porcine parvoviruses (PPV) have been identified, classic PPV (PPV1), PPV2, PPV3 (known as porcine PARV4, hokovirus, or partetravirus), PPV4, and porcine bocaviruses, which all have substantial genetic divergence (1–5).

In the present study, a new PPV was discovered. Initially four DNA fragments (704 bp) with an identity of about 76% to the known sequences of PPV4 were identified by primers used for screening porcine lung samples randomly selected among routine submissions to the Iowa State University Veterinary Diagnostic Laboratory. The rather low identity indicated a possible novel PPV4 subtype or a new PPV closely related to PPV4. Further genome sequencing was carried out by utilizing modified sequence-independent single-primer amplification (6) followed by primer walking. The 5′ and 3′ ends of the genome were obtained by inverse PCR, which was used to amplify the circular or head-to-tail concatameric templates as described for PPV4 (3). The PCR products were cloned and sequenced, with the sequence contigs assembled by using the software DNAMan version 7 (Lynnon Corporation). A parvovirus genome possessing a circular or head-to-tail array was identified that showed the closest relationship to PPV4, with overall identities of 64.1 to 67.3% to available PPV4 genome sequences. According to the existing criteria of parvovirus species classification defined as <95% related by nonstructural gene DNA by the International Committee on Taxonomy of Viruses (1), this virus was considered a novel species and tentatively designated PPV5.

The genome of PPV5 is comprised of 5,805 bp, with a GC content of 40.9%, similar to other PPVs. Two major putative ORFs (ORF1 and ORF2) were predicted, indicating a genome organization similar to PPV1, PPV2, and PPV3, but different from PPV4, which contains an additional ORF3 similar to bocavirus (3, 7). The predicted ORF1 (bp 862 to 2667) and ORF2 (bp 2787 to 5762) encode proteins of 601 and 991 amino acids, which are 3 and 263 amino acids longer than ORF1 (598 amino acids) and ORF2 (728 amino acids) of PPV4, respectively. Moreover, the nucleotide and amino acid sequences of NS (ORF1) showed identities of 77.4% to 78.2% and 84.6% to 85.1%, and the nucleotide and amino acid sequences of the capsid (ORF2) showed low identities of 59.2% to 60.1% and 54.0% to 54.3% to those of the closest related PPV4, respectively, indicating distinct genetic characteristics of PPV5. Further studies on the epidemiology and pathogenesis of PPV5 are under way.

### Nucleotide sequence accession numbers. 

The genome sequences of the PPV5 strains IA469-clone1, IA469-clone2, IN273, MI216, and ND564 have been deposited in GenBank under the accession numbers JX896318 through JX896322. The PPV5 described here corresponds to IA469-clone1 (JX896321).
